# An Automated Population Monitoring Framework for *Larus ridibundus* in Kunming City Based on Improved YOLOv8 and ByteTrack

**DOI:** 10.3390/s26154948

**Published:** 2026-08-05

**Authors:** Yonglin Che, Yucheng Zeng, Zhaoxiang Ma, Qian Xia, Rongxin Zhang, Lichang Chen, Jiajin Zhang, Hanming He

**Affiliations:** 1 College of Big Data, Yunnan Agricultural University, Kunming 650201, China; 2024240737@stu.ynau.edu.cn (Y.C.); 2024240731@stu.ynau.edu.cn (Y.Z.); 2025210564@stu.ynau.edu.cn (Z.M.); 2025240790@stu.ynau.edu.cn (Q.X.); 2025240775@stu.ynau.edu.cn (R.Z.); 2 College of Mechanical and Electrical Engineering, Yunnan Agricultural University, Kunming 650201, China

**Keywords:** *Larus ridibundus*, recognition and counting, deep learning, YOLOv8n, ByteTrack

## Abstract

The *Larus ridibundus* (*L. ridibundus*), a prominent part of Kunming’s landscape, attracts many tourists and boosts the local tourism industry. Effective population monitoring of this species matters for wetland environment evaluation, biodiversity conservation, and ecological civilization construction. Currently, *L. ridibundus* population statistics mainly rely on manual methods, which are labor-intensive and inefficient. To overcome these limits, we propose a deep learning (DL) framework. It automatically recognizes and counts *L. ridibundus* by combining CDSP2-YOLOv8n with ByteTrack, aiming to efficiently monitor their population metrics. Our framework uses the optimized YOLOv8n model to achieve excellent multi-object detection for this species. It also uses ByteTrack to effectively reduce target loss from occlusion or overlap during the birds’ flight, providing a sophisticated DL approach for population monitoring. Experimental results show the modified CDSP2-YOLOv8n model works well on the collected *L. ridibundus* multi-object detection dataset. Its mAP@0.5, mAP@0.5:0.95, Precision, and Recall reach 0.9705, 0.6557, 0.9685, and 0.9496, respectively. When combined with ByteTrack, the proposed framework achieved a Multiple Object Tracking Accuracy (MOTA) of 89.7% and a Multiple Object Tracking Precision (MOTP) of 83.5%. It also demonstrated superior performance in terms of IDF1, Mostly Tracked (MT), Mostly Lost (ML), and Identity Switches (IDSs). Compared to manual counting, our framework has an average accuracy of 91.58%, greatly enhancing the efficiency and accuracy of *L. ridibundus* population monitoring. In summary, we successfully achieved the automated recognition and counting of *L. ridibundus*. The proposed method accurately identifies and consistently tracks individual birds, enabling effective population counting. It provides a novel and comprehensive technical approach for the monitoring and conservation of this species and demonstrates promising potential for practical applications.

## 1. Introduction

Growing awareness of ecological conservation has made wildlife protection and monitoring critical topics among scholars worldwide [1]. Each winter, the aggregation of *L. ridibundus* in Kunming not only enhances the local landscape but also attracts thousands of tourists. This phenomenon promotes ecological tourism and strengthens public engagement with nature conservation. However, urbanization has accelerated environmental pollution, habitat fragmentation, and human interference, posing significant threats to *L. ridibundus*’ survival and reproduction. Because *L. ridibundus* is a key ecological indicator, its population trends directly reflect the health of the ecosystem. Monitoring their population dynamics enables protected area managers to assess survival and distribution patterns, guiding effective conservation strategies. Thus, efficient monitoring is essential to addressing *L. ridibundus*’ conservation challenges.

Traditional avian monitoring primarily depends on manual observation combined with specialized equipment. Ornithologists and researchers typically utilize tools such as telescopes, high-resolution cameras, and telephoto lenses to conduct focused observations in bird habitats [2,3]. However, this approach is not only time-consuming and labor-intensive but also constrained by limited coverage, often leading to delayed, inaccurate, and incomplete data. Consequently, there is a pressing need for automated technologies to enhance the precision of *L. ridibundus* monitoring.

The rapid advancement of computer vision (CV) and artificial intelligence (AI) has facilitated the application of interdisciplinary research methods for detecting plants and animals [4,5,6,7]. Notably, object detection algorithms such as YOLO [8,9,10,11] and Faster R-CNN [12], alongside tracking algorithms like SORT [13], DeepSORT [14,15], and ByteTrack [16], have been successfully employed for dynamic species counting, including fish [17,18,19], sheep [5,20], chickens [21], and pigs [22,23,24]. In contrast to traditional manual observation and counting, these methods offer superior robustness, adeptly managing challenges such as variable lighting, distinct backgrounds, and changes in camera angles. They also deliver enhanced computational efficiency and maintain high accuracy across diverse experimental conditions. While these methods were not specifically designed for *L. ridibundus* recognition and counting, they provide a solid technological foundation and valuable research methodologies for the study presented in this paper.

In ornithological research, numerous scholars have endeavored to integrate bird recognition algorithms with tracking techniques to enhance the detection and tracking accuracy of avian species [25,26,27]. These methods typically leverage DL and CV technologies, combining the strengths of object detection and tracking to enable precise bird monitoring in complex environments. However, birds in flight often pose significant challenges, such as small size, high density, and occlusion [28,29], which hinder the efficacy of existing algorithms in real-world applications. Consequently, real-time detection and tracking of aerial bird targets remain a persistent challenge, both now and for the foreseeable future.

In the automatic and precise recognition and counting of *L. ridibundus*, similar challenges arise, such as small target size, high density, and limited research in this area. While existing counting algorithms share structural similarities, the flight patterns, morphological features, density, and camera angles of *L. ridibundus* differ from those of other species. Consequently, current general-purpose algorithms are not directly applicable to *L. ridibundus* counting tasks. Specifically, in video tracking, there is a lack of accurate, versatile, and easily deployable counting algorithms for *L. ridibundus*. Therefore, to effectively apply these algorithms for counting *L. ridibundus* in Kunming’s natural habitats, optimization based on the unique behavioral traits of gulls and habitat complexity is necessary. Such optimization is crucial for enhancing algorithm accuracy and stability, while addressing challenges like high density and occlusion.

Overall, monitoring the population of *L. ridibundus* is critical for its conservation and ecological protection. To strengthen the protection of this species, the contributions of this study are as follows:

(i) Creation of a comprehensive *L. ridibundus* dataset. This dataset contains 2170 image frames of *L. ridibundus* captured via mobile phone, alongside 38 video clips of the species.
(ii) Development of CDSP2-YOLOv8n. Using YOLOv8n as the baseline model, CDSP2-YOLOv8n was constructed to effectively address the challenges of small target sizes and rapid movement, thereby enhancing the detection accuracy of *L. ridibundus* under diverse environmental conditions.
(iii) Algorithm integration. The optimized architecture is combined with the ByteTrack tracking algorithm for the identification and tracking of *L. ridibundus*. The high-precision CDSP2-YOLOv8n model reduces false positives and missed detections, while the ByteTrack algorithm effectively manages the counting challenges caused by overlapping gulls.
(iv) System construction and verification. An *L. ridibundus* tracking and counting system based on CDSP2-YOLOv8n and the ByteTrack algorithm was developed. Extensive experiments have verified that this system can perform accurate counting of *L. ridibundus*.

## 2. Materials and Methods

### 2.1. Data Collection

The data collection for this study was conducted in Cuihu Park, Daguan Park, and the Haiceng Dam area in Kunming—common habitats for *L. ridibundus*, exhibiting notable ecological diversity. During the collection, various environmental conditions and gull activity states, such as lighting, weather fluctuations, and group aggregation or dispersion, were considered. Potential interference factors, including overlapping gulls, distance variations, and obstructions, were minimized to ensure data representativeness and diversity.

The data were gathered from October 2024 to March 2026 using a Vivo X100S smartphone (manufactured by Vivo Mobile Communication Co., Ltd., Dongguan City, Guangdong Province, China) to capture images. A total of 894 images (4032 × 3024 resolution) and 38 MP4-format videos of *L. ridibundus* were recorded, with a cumulative video duration of 3 h and 51 min at 30 frames per second. To reduce annotation costs while maintaining data representativeness, one frame was extracted every 20 frames from the videos. Using OpenCV, the videos were processed frame by frame, yielding 1276 images (4032 × 3024 resolution) in JPG format. The final dataset comprised 2170 images, including both original still images and video-derived frames, which were used for *L. ridibundus* object detection. To ensure a reliable evaluation, video-derived images were subsequently partitioned according to their original video sequences during dataset splitting.

### 2.2. Data Preprocessing

#### 2.2.1. Data Annotation and Dataset Splitting

The positions of *L. ridibundus* in the images were labeled using LabelImg (v1.8.1) software in this study. The specific method involved marking the positions of the gulls and drawing rectangular bounding boxes around them on each image to generate the corresponding annotation files. Each file records the location information of the *L. ridibundus*, including the coordinates of the four vertices of the bounding box, as well as other related information such as the path and the image name.

After annotation, the dataset was divided into training, validation, and test sets at a ratio of 8:1:1. For images extracted from videos, a video-level splitting strategy was adopted, where all frames originating from the same video sequence were assigned exclusively to one subset. The original still images were then distributed accordingly. This strategy prevented temporally correlated samples from different subsets and reduced the risk of performance overestimation caused by temporal leakage.

#### 2.2.2. Image Augmentation

During the training of DL models, insufficient training data may result in overfitting, thereby compromising the model’s capacity for generalization. To enhance the model’s performance and robustness, data augmentation strategies are employed to generate a wider array of training samples, enabling the model to acquire data features from multiple viewpoints.

**Figure 1:**
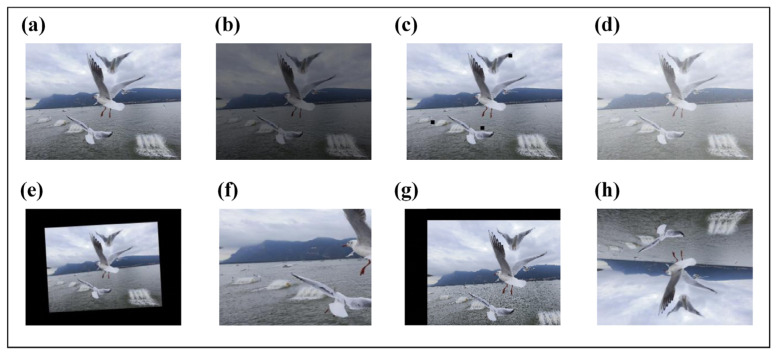
Comparison of images before and after data augmentation. Image (**a**) shows the original input, while (**b**–**h**) represent images augmented via brightness adjustment, random occlusion, gaussian noise, rotation, cropping, translation, and mirror flipping, respectively.

In this study, seven data augmentation techniques were applied to the training set to increase the number of training samples by a factor of four, while the validation and test sets remained unchanged. The augmentation methods included brightness transformation, random occlusion, Gaussian noise, rotation, random cropping, translation, and horizontal flipping. As a result, the size of the dataset increased from 2170 to 8680 images. Examples of the augmented images are presented in Figure 1.

### 2.3. CDSP2-YOLOv8n Network Architecture

**Figure 2:**
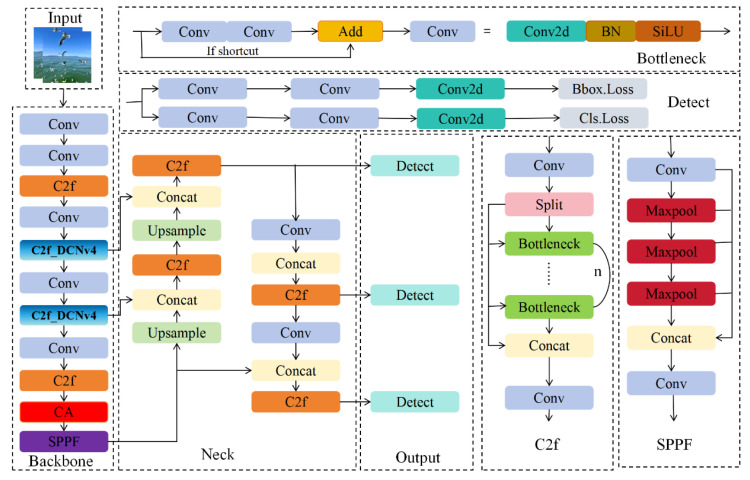
CDSP2-YOLOv8n network architecture.

To improve the monitoring performance of *L. ridibundus* in complex natural environments, this study proposes a task-oriented object detection framework, termed CDSP2-YOLOv8n, based on YOLOv8n. The proposed method adopts a collaborative optimization strategy to address the unique characteristics of *L. ridibundus*, including high target density, small object size, rapid flight, and frequent occlusion. Rather than functioning as independent modifications, the proposed components are jointly designed and optimized to complement one another, enabling collaborative improvements in feature representation, geometric modeling, difficult-sample optimization, and multi-scale object perception. Through this unified optimization strategy, the overall detection performance in complex scenarios is effectively enhanced. Specifically, as illustrated in Figure 2, YOLOv8n is adopted as the baseline detector and systematically optimized from multiple perspectives. First, the Coordinate Attention (CA) mechanism [30] and the C2f_DCNv4 module are incorporated into the backbone network to enhance discriminative feature extraction and improve the adaptive modeling capability for geometric deformations of small objects. Second, the original CIoU Loss is replaced with Slide Loss to achieve a more balanced optimization of easy and hard samples during training, thereby improving the robustness of the detector across different monitoring scenarios [31].

Furthermore, because *L. ridibundus* generally occupy only a small proportion of the image, the detection head of YOLOv8n was redesigned. A P2 detection layer was introduced to enhance fine-grained feature representation for small objects, while the P5 detection layer was removed because large-object detection is less relevant to the present task. This structural adjustment partially offsets the additional parameter and computational overhead introduced by the high-resolution P2 branch.

These improvements effectively enhance the performance of CDSP2-YOLOv8n for the multi-object detection of *L. ridibundus*. The following subsections provide a detailed description of each proposed improvement.

#### 2.3.1. Baseline Model

The YOLO series is regarded as one of the most stable object detection frameworks today. It began with YOLOv1 [8] in 2015, which introduced the foundational concept of a single-stage detection algorithm, substantially accelerating detection speed while preserving high detection accuracy, thereby addressing the slow inference challenges inherent in two-stage detection models. Subsequently, YOLOv2 [32] incorporated joint training techniques, enabling the detection of over 9000 object classes. YOLOv3 [33] further refined the architecture by integrating Darknet-53 and FPN architectures, facilitating multi-scale prediction and feature fusion, and introducing batch normalization and spatial pyramid pooling (SPP) modules, enhancing detection precision. From YOLOv4 [34] to YOLOv7 [35], major advancements were made in model architecture and training methodologies, augmenting gradient flow and feature extraction capabilities, and boosting adaptability to multi-scale objects.

YOLOv8 [36,37] represents another pivotal advancement in the YOLO series, revolutionarily integrating the expanded PaFPN framework within the YOLOv5 architecture. Furthermore, YOLOv8 adopts a cascading model scaling methodology, dynamically generating the most optimal model variant (n, s, m, l, x) tailored to specific task requirements, thereby amplifying both robustnessand versatility. These breakthroughs enable YOLOv8 to outperform previous iterations in terms of both speed and precision, establishing it as an indispensable asset in the realm of object detection.

Although the YOLO series has recently progressed to its latest iterations, such as YOLO26 [38], these newer versions exhibit certain limitations. Their underlying model architectures are subject to rapid changes, the cross-platform deployment toolchains designed for low-power edge computing devices in field settings remain in the developmental stage, and they lack long-term engineering validation for ecological monitoring in complex natural environments. In contrast, YOLOv8 has undergone extensive community validation, establishing itself as a remarkably stable version with a mature and compatible deployment ecosystem, supported by frameworks such as TensorRT, ONNX, and OpenVINO. Therefore, the lightweight YOLOv8n was selected as the baseline framework, and targeted optimizations were performed to improve its detection performance for *L. ridibundus* across different scenarios in Kunming.

#### 2.3.2. CA Mechanism

**Figure 3:**
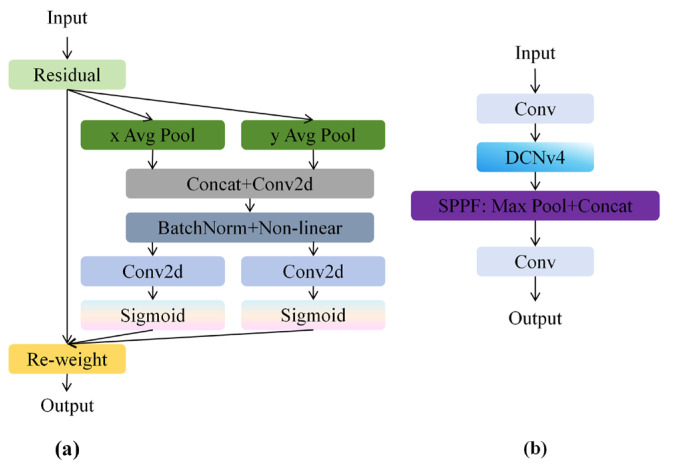
Structures of CA and C2f_DCNv4. (**a**) illustrates the module structure of CA, and (**b**) illustrates the module structure of C2f_DCNv4.

Attention mechanisms [39] have been extensively applied in DL to improve model accuracy and computational efficiency. In this work, we enhanced the performance of CDSP2-YOLOv8n by integrating a CA module (Figure 3a), with the specific insertion location details shown in Figure 2.

The CA mechanism [30] enhances the DL model’s ability to extract spatial features, especially in resource-constrained environments. Its key advantage lies in its ability to enhance the model’s perception of long-range dependencies while maintaining high computational efficiency. The mechanism encodes spatial information using global pooling and generates one-dimensional coordinate features, helping the model better capture dependencies across different spatial locations.

The CA mechanism works by introducing coordinate information to improve spatial feature extraction, particularly in environments with limited resources. The input is typically a feature map from a convolutional neural network at a certain layer, with the dimensions [*C*, *H*, *W*], where *C* represents the number of channels, which refers to the different feature channels in the feature map; *H* represents the height of the feature map, which corresponds to the vertical dimension; *W* represents the width of the feature map, corresponding to the horizontal dimension.

The core operations of this mechanism involve three main steps. The first is the information embedding operation, where the feature map is globally pooled along the horizontal and vertical directions. This produces a feature map that contains positional information, aiding in the extraction of coordinate information from the image space and supporting the subsequent attention mechanism. The next operation is attention generation, where the pooled feature map is concatenated and passed through a 1 × 1 convolution followed by an activation function, resulting in feature maps that represent spatial information. These maps generate attention vectors in both the horizontal and vertical directions. The final step is featuring map correction, where the generated attention vectors are multiplied by the input feature map, allowing for weighted adjustments in specific regions to enhance or suppress certain features.

Ultimately, the CA mechanism optimizes the feature extraction process, allowing the model to better capture spatial relationships. The primary computational formula for CA is:

(1) $$\begin{array}{c} y_{c}\left(i,j\right)=x_{c}\left(i,j\right)\times g_{c}^{h}\left(i\right)\times g_{c}^{w}\left(j\right) \end{array}$$

where $g_{c}^{h}\left(i\right)$ and $g_{c}^{w}\left(j\right)$ are the attention vectors in the horizontal and vertical directions, respectively.

#### 2.3.3. C2f_DCNv4 Module

Traditional convolution employs kernels with fixed sizes and extracts local features by sliding over the input feature map with an invariant receptive field. However, in practical object detection tasks, objects exhibit significant variations in scale, shape, and spatial distribution. Consequently, the fixed sampling locations of conventional convolution are insufficient to effectively capture geometric structural information, particularly for small objects, resulting in inadequate feature representation and reduced localization accuracy. To enhance the feature modeling capability of CDSP2-YOLOv8n for complex object detection scenarios, this paper introduces the DCNv4 module [40] and constructs the c2f_DCNv4 module (Figure 3b), which is integrated into the CDSP2-YOLOv8n architecture (the insertion locations are shown in Figure 2). By dynamically adjusting the sampling locations of the convolution kernels, the DCNv4 component within C2f_DCNv4 enables the network to adaptively perceive the geometric deformations and scale variations in the targets.

Specifically, to overcome the limitations of conventional convolution, DCNv4 extends the standard convolution by introducing learnable spatial offsets and unbounded dynamic modulation weights, enabling the sampling locations of the convolution kernel to be adaptively adjusted according to the input features. The mathematical formulation of DCNv4 is expressed as follows:

(2) $$\begin{array}{c} y\left(P_{0}\right)=\sum_{K=1}^{K}w_{K}m_{K}x\left(P_{0}+P_{K}+{∆P}_{K}\right) \end{array}$$

where *P*_0_ denotes the current output location, *y* represents the output feature at position *P*_0_, and *x* denotes the input feature sampled at the corresponding location. *K* is the total number of sampling points in the convolution kernel, *W_K_* is the convolution weight associated with the *K*-th sampling point, and *P_K_* represents the predefined relative sampling position in standard convolution. The learnable offset Δ*P_K_* dynamically adjusts the sampling location, and bilinear interpolation is employed to estimate the feature value when the sampling coordinates are fractional. In addition, *m_K_* denotes the dynamic modulation weight of the *K*-th sampling point, which adaptively determines the contribution of each sampled feature to the final convolution output.

As seen from Equation (2), DCNv4 can adaptively adjust the importance of each sampling point based on the distribution of the input features, thereby further enhancing the network’s capability to represent geometrically deformed objects and fine-grained features. The C2f_DCNv4 module effectively utilizes this characteristic. When integrated into the CDSP2-YOLOv8n network, it enables the convolution kernels to dynamically optimize their sampling locations according to the actual shape and spatial distribution of the target objects. This improvement significantly boosts the model’s ability to extract discriminative features from tiny objects, irregularly shaped targets, and objects in complex scenes, thereby effectively enhancing the detection accuracy of *L. ridibundus*.

#### 2.3.4. Slide Loss Function

Slide Loss [41] is a loss function tailored for object detection, offering distinct advantages. Its key feature is the adaptive weighting mechanism, which dynamically adjusts the weights for challenging targets, enhancing detection accuracy for distant, overlapping, and boundary-obscured objects, while also minimizing false positives and negatives. In contrast to conventional loss functions, Slide Loss is more robust in complex environments, such as water reflections and branch occlusions, contributing to model stability. Furthermore, the introduction of a sliding window mechanism mitigates overfitting risks during bounding box regression, yielding more precise target position and shape predictions.

Slide Loss primarily distinguishes between easy and hard samples based on the Intersection over Union (IoU) between the predicted and ground truth boxes. IoU serves as a measure of the overlap between the predicted and ground truth boxes: a higher IoU indicates a better match and more accurate detection, while a lower IoU suggests a larger deviation from the true target, indicating a hard sample. The weighted function of Slide Loss is defined as follows in Equation (3).

(3) $$\begin{array}{c} f\left(x\right)=\left\{\begin{array}{cc} 1, & x\leq \mu -0.1\\ e^{1-\mu }, & \mu <x<\mu -0.1\\ e^{1-x}, & x\geq \mu \end{array}\right. \end{array}$$

where *x* represents the IoU value of the sample and *μ* represents the mean IoU of all samples.

In the task of detecting *L. ridibundus*, the density of bird flocks varies significantly, and the targets exhibit considerable scale disparity. Conventional methods often struggle to detect small or distant targets effectively. However, Slide Loss applies adaptive weighting to gulls of varying sizes, thereby directing the model’s focus towards smaller targets during training, thereby enhancing overall detection accuracy.

#### 2.3.5. Small Object Detection Head Design

To address the challenge of detecting objects at different scales, the YOLOv8 network employs three detection layers, namely P3, P4, and P5, with feature map sizes of 80 × 80, 40 × 40, and 20 × 20, respectively, for detecting small, medium, and large objects. For an input image of 640 × 640 pixels, the Backbone and Neck networks are responsible for feature extraction, while the final object detection is performed by the Head. The three detection layers operate on feature maps with downsampling factors of 8×, 16×, and 32×, respectively.

However, in this study, the target objects, *L. ridibundus*, are relatively small. The large downsampling factors employed by the original YOLOv8 network cause the deep feature maps to lose fine-grained information, making them less effective for representing small objects. As a result, objects smaller than 8 × 8 pixels are prone to missed detections and false detections. To overcome this limitation, a P2 detection layer is introduced into the CDSP2-YOLOv8n architecture to improve the detection of smaller-scale targets.

Specifically, in the Feature Pyramid Network (FPN), the 80 × 80 feature map is first upsampled to 160 × 160 and then fused with the corresponding shallow feature map from the Backbone to enhance the representation of fine-grained details for small objects. Meanwhile, a corresponding downsampling path is incorporated into the Path Aggregation Network (PAN) to maintain feature flow consistency and facilitate multi-scale feature fusion. Furthermore, the P5 detection branch was removed to partially offset the additional computational complexity introduced by the P2 branch. As a result, the redesigned detection head consists of three detection scales, namely P2, P3, and P4. Compared with the four-scale P2–P5 configuration, this design reduces the number of parameters and computational complexity while improving the detection performance for very small objects.

### 2.4. L. ridibundus Multi-Target Tracking Algorithm Design

During the multi-object tracking (MOT) stage of *L. ridibundus*, ByteTrack [16] was adopted as the object tracking algorithm. ByteTrack is a tracking-by-detection-based MOT framework that associates both high-confidence and low-confidence detection boxes instead of directly discarding low-confidence detections. By fully exploiting valid detection information with different confidence levels, ByteTrack effectively alleviates missed detections and trajectory fragmentation caused by object occlusion, motion blur, and detection confidence degradation, thereby improving trajectory continuity and identity (ID) consistency.

**Figure 4:**
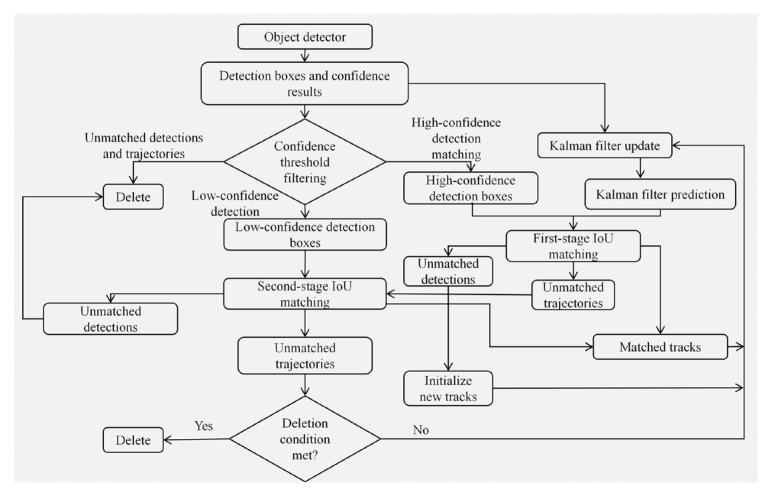
ByteTrack tracking algorithm workflow.

The overall workflow of ByteTrack is illustrated in Figure 4. First, the CDSP2-YOLOv8n detector generates bounding boxes and corresponding confidence scores for each input video frame. The detection results are processed through confidence-based filtering and divided into high-confidence and low-confidence detection sets. Meanwhile, the Kalman filter predicts the states of existing trajectories and estimates the object locations in the current frame. Subsequently, ByteTrack performs a two-stage association strategy based on IoU similarity. In the first stage, high-confidence detections are associated with predicted trajectories generated by the Kalman filter. The matched detections are used to update the corresponding trajectories, while unmatched detections and trajectories are retained for further association. In the second stage, unmatched trajectories are further associated with low-confidence detections. Unlike conventional tracking algorithms that discard low-confidence detections, ByteTrack utilizes these potential object observations to recover targets affected by temporary occlusion, motion blur, or reduced detection confidence. This strategy improves trajectory continuity and reduces identity switches under complex monitoring conditions. After the two-stage association process, unmatched high-confidence detections are used to initialize new trajectories, whereas trajectories that remain unmatched for multiple consecutive frames are removed. Through the combination of Kalman filter prediction, confidence-aware detection association, and IoU-based matching, ByteTrack generates stable trajectories with unique identities, providing reliable identity information for subsequent population counting.

In this study, ByteTrack was integrated with the CDSP2-YOLOv8n detector through the Ultralytics tracking framework. The detection confidence threshold was set to 0.15, and the non-maximum suppression (NMS) IoU threshold was set to 0.45. The persistent tracking mode was enabled (persist = True) to maintain trajectory information between consecutive frames. The main ByteTrack association parameters were configured as follows: track_high_thresh = 0.25, track_low_thresh = 0.10, new_track_thresh = 0.25, track_buffer = 30, match_thresh = 0.80, and fuse_score = True.

### 2.5. L. ridibundus Counting Algorithm Design

To achieve reliable population estimation, a full-frame region-based counting strategy was adopted, as shown in Algorithm 1. The entire video frame was considered as the counting region for each video sequence, as all visible *L. ridibundus* individuals within the camera field of view were included in the observation area. During counting, each detected gull was assigned a unique tracking ID by ByteTrack. When a new gull trajectory appeared in the video sequence, its corresponding tracking ID was recorded and counted once. If the same tracking ID appeared again in subsequent frames, it was ignored to prevent duplicate counting caused by continuous observation of the same individual, temporary occlusion, or repeated appearance. This strategy ensures that each individual *L. ridibundus* appearing in the video sequence is counted only once based on its unique trajectory identity.
**Algorithm 1** MOT and counting method for *L. ridibundus***Input:** CDSP2-YOLOv8n detector; ByteTrack tracker; video frame sequence *F*.
Output: Total count of *L. ridibundus* (*C*).
**1.** **Phase 1: Initialize****2.** *C* ← 0, *ID_recorded*← θ**3.** **Phase 2: Detection and tracking****4.** for *i* ← 1 to *N* in *F* do**5.** *D_i_* ← CDSP2-YOLOv8n (*F_i_*)**6.** *T_i_* ← ByteTrack (*D_i_*)**7.** *A* ← ones_like (*F_i_*)**8.** **Phase 3: Counting****9.** The entire video frame is defined as the counting region**10.** for each detected *L. ridibundus L_i_* in *T_i_* do**11.** *P_i_* ← center position of *L_i_***12.** *ID_i_* ← tracking *ID* of *Li***13.** if *A*[*P_i_*] == 1 then
**14.** 
if *ID_i_* ∉ *ID_recorded* then**15.** *ID_recorded*.add (*ID_i_*)**16.** *C* + ← 1**17.** **Return** *C*

## 3. Results

### 3.1. Experimental Environment

The experiments were conducted on a system running the Windows 11 operating environment, utilizing the PyTorch DL framework. The Python version employed was 3.8, with PyTorch 1.9.0 and CUDA 11.1 for computational support. The hardware configuration comprised an Intel(R) Core(TM) i9-10900KF CPU, operating at a base clock speed of 3.70 GHz, alongside an NVIDIA GeForce RTX 3080 graphics card, equipped with 10 GB of dedicated memory.

To ensure a fair comparison, all models were trained and evaluated using the same dataset partition, including identical training, validation, and testing subsets. The same preprocessing strategy, data augmentation pipeline, input resolution (640 × 640), training epochs (300 epochs), batch size (16), and evaluation metrics were adopted for all comparative models. All models were trained from scratch without using pretrained weights. Furthermore, all experiments were conducted under identical hardware and software environments, with the random seed fixed to 42 to reduce the influence of random initialization and improve reproducibility. These controlled settings minimize experimental bias caused by different training conditions and enable a more reliable comparison of model performance.

### 3.2. Evaluation Metrics

To scientifically evaluate the robustness of the CDSP2-YOLOv8n object detection algorithm, we employed several commonly used evaluation metrics: average precision at different confidence thresholds (*mAP*@0.5, *mAP*@0.5:0.95), *Precision*, *Recall*, as well as *Parameters* (*Params*) and computational complexity (*GFLOPs*).

(4) $$\begin{array}{c} Precision=\frac{TP}{TP+FP} \end{array}$$

(5) $$\begin{array}{c} Recall=\frac{TP}{TP+FN} \end{array}$$

where *TP* represents the number of *L. ridibundus* targets successfully detected by the model, *FP* represents the number of false positives (incorrectly identified targets), and *FN* represents the number of false negatives (missed targets).

*AP* reflects the model’s detection accuracy at different recall rates. *mAP* is the average of *AP* across all classes. Since this study focuses on a single target class, the *L. ridibundus*, *mAP* is equivalent to *AP* in this case. The calculation formula for *AP* is given in Equation (6).

(6) $$\begin{array}{c} AP=\int_{0}^{1}P\left(R\right)d\left(R\right) \end{array}$$

where *P*(*R*) represents the precision at a given recall rate *R*.

Furthermore, MOTA, MOTP, IDF1, MT, ML, and IDS, and Frames Per Second (FPS) were used to evaluate overall tracking performance, localization consistency for correctly matched targets, and computational efficiency, respectively. The main calculation formula is as follows:

(7) $$\begin{array}{c} MOTA=1-\frac{\sum_{t}\left(FN_{t}+FP_{t}+IDS_{t}\right)}{\sum_{t}{GT}_{t}} \end{array}$$

where *FN_t_* is the number of missed targets at time *t*, *FP_t_* is the number of false detections, *IDS_t_* is the number of *ID* switches, and *GT_t_* is the number of true targets at time *t*.

(8) $$\begin{array}{c} MOTP=\frac{\sum_{t,i}d_{t,i}}{\sum_{t}c_{t}} \end{array}$$

where *d_t,i_* is the matching error between target *i* at time *t*, and *c_t_* is the number of correctly detected targets at time *t*.

(9) $$\begin{array}{c} IDF1=\frac{2IDTP}{2IDTP+IDFP+IDFN} \end{array}$$

where *IDTP* is the number of correctly identified detections, *IDFP* is the number of detections assigned to incorrect identities, and *IDFN* is the number of ground-truth detections whose identities were missed or incorrectly matched.

(10) $$\begin{array}{c} MT=\frac{N_{MT}}{N_{traj}} \end{array}$$

where *N_MT_* is the number of ground-truth trajectories successfully tracked for at least 80% of their lifespan, and *N_traj_* is the total number of ground-truth trajectories.

(11) $$\begin{array}{c} MT=\frac{N_{ML}}{N_{traj}} \end{array}$$

where *N_ML_* is the number of ground-truth trajectories successfully tracked for no more than 20% of their lifespan.

(12) $$\begin{array}{c} IDS=\sum_{t=1}^{T}IDS_{t} \end{array}$$

where *IDS_t_* is the number of identity-switching events at time *t*. An identity switch is recorded when a ground-truth target is assigned a predicted identity different from that associated with its previous valid match. A lower IDS value indicates better identity consistency during tracking.

(13) $$\begin{array}{c} FPS=\frac{1}{T} \end{array}$$

where *T* is the average time required for the algorithm to process a single frame.

### 3.3. Experimental Results of Adding Attention Mechanism

**Figure 5:**
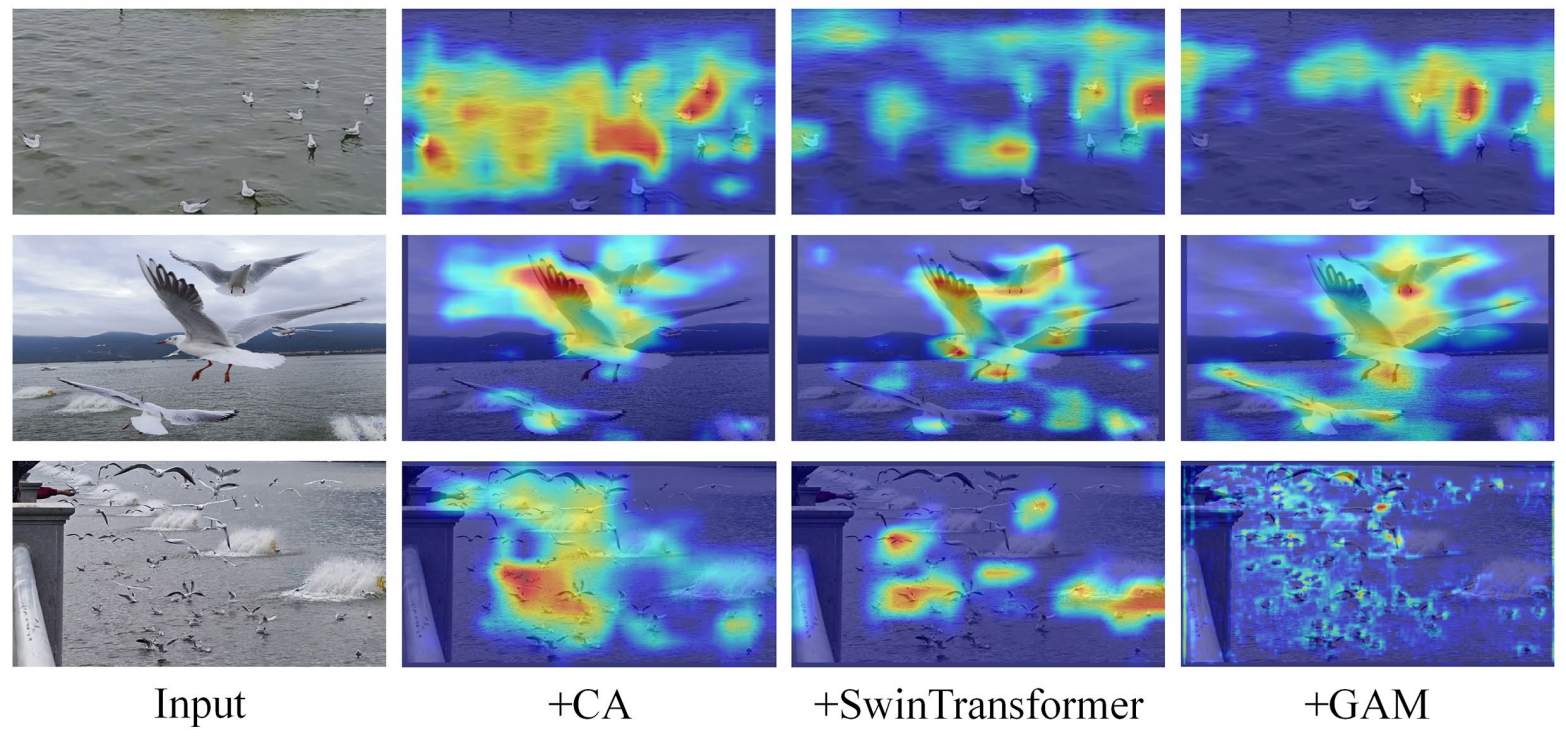
Shows the heatmap visualization after introducing different attention mechanisms into YOLOv8.

To evaluate the impact of different attention mechanisms on network robustness, we integrated CA [30], Swin Transformer [42], and GAM [43] mechanisms into the YOLOv8 network model and conducted comparative experiments. Table 1 presents the comparison results of the YOLOv8n_CA, YOLOv8n_Swin Transformer, and YOLOv8n_GAM models against the base YOLOv8n model across four different metrics, while Figure 5 illustrates their heatmap visualization performance.

Compared to the base YOLOv8n model, the introduction of the GAM attention mechanism led to a noticeable enhancement in performance. Specifically, the mAP@0.5, mAP@0.5:0.95, Precision, and Recall increased by 0.94%, 3.9%, 1.43%, and 1.5%, respectively. However, it also required higher computational resources, with an increase of 1.64 M in parameters and an additional 1.3 G in computation. The YOLOv8n_CA model showed advancements in mAP@0.5:0.95, mAP@0.5, Precision, and Recall by 0.8%, 2.52%, 0.93%, and 1.66%, respectively. The increase in parameters and computational complexity was minimal, with a rise of only 0.04 M in parameters and 0.1 G in computation. In contrast, the YOLOv8n_Swin Transformer model’s effectiveness in terms of mAP@0.5:0.95, mAP@0.5, Precision, and Recall was similar to that of YOLOv8n_CA, but it significantly increased the computational load. It required 10.4 G more computation than YOLOv8n, 10.3 G more than YOLOv8n_CA, and 9.1 G more than YOLOv8n_GAM. The parameter increase was 0.36 M more than YOLOv8n and 0.32 M more than YOLOv8n_CA, but 1.28 M less than YOLOv8n_GAM.

Through a comprehensive comparison and analysis, it was found that while the YOLOv8n_GAM model achieved the highest overall accuracy, it also incurred a substantial increase in parameters and computational load. In contrast, the integration of the CA attention mechanism into the YOLOv8n model led to significant advancements in *L. ridibundus* target recognition accuracy, all while preserving relatively low computational demands. Consequently, the CA attention mechanism was selected to optimize the network architecture of the YOLOv8n model.

### 3.4. Ablation Experiment

#### 3.4.1. Ablation Study on the Small Object Detection Layer

The ablation study analyzing the impact of the small object detection layer on model efficiency is presented in Table 2. The YOLOv8n_P2345 model demonstrated enhancements in mAP@0.5, mAP@0.5:0.95, Precision, and Recall by 1.88%, 2.49%, 0.49%, and 3.56%, respectively, compared to the YOLOv8n model. Although the parameter count decreased marginally by 0.08 M, the computational complexity increased significantly, with an additional 4.2 G due to the inclusion of the P2 detection layer, which focuses on detecting small targets (4 × 4). This allows the model to extract finer-grained features, but requires additional network components, leading to higher accuracy at the cost of increased computational demand.

In the YOLOv8 architecture, the P5 detection layer, responsible for large target detection, occupies a substantial portion of the network. To evaluate its impact on model accuracy and computational load, the 20 × 20 large target detection layer was removed from the backbone, facilitating stronger fusion of shallow and deep features. The results showed that, compared to YOLOv8n_P2345, the YOLOv8n_P234 model improved mAP@0.5, mAP@0.5:0.95, Precision, and Recall by 0.32%, 0.73%, 0.53%, and 0.4%, respectively, and by 2.2%, 3.22%, 1.02%, and 3.96% compared to YOLOv8n.

In terms of resource usage, the YOLOv8n_P234 model reduced the number of parameters by 0.92 M and computation by 0.8 G compared to YOLOv8n_P2345. However, compared to YOLOv8n, the parameter count decreased by 1 M, but computation slightly increased by 3.4 G.

The experimental results indicate that this adjustment effectively reduces the model’s parameter count while maintaining detection accuracy.

#### 3.4.2. Ablation Study of Individual Components

To evaluate the contribution of each proposed component, including the CA mechanism, C2f_DCNv4 module, Slide Loss function, and P2 small object detection layer, a series of ablation experiments were conducted. Unlike a simple cumulative comparison, the experiments were designed to separately investigate the effect of each individual module and their interactions. The quantitative results are presented in Table 3. Experiment 1 represents the baseline YOLOv8n model. Experiments 2–5 introduce each proposed component individually, while Experiments 6–8 evaluate the combined effects of different modules.

Compared with the baseline model (Experiment 1), introducing the CA attention mechanism (Experiment 2) improves mAP@0.5 from 0.9477 to 0.9557 and mAP@0.5:0.95 from 0.6120 to 0.6372, accompanied by improvements in Precision and Recall. These results demonstrate that the CA mechanism effectively enhances feature representation by adaptively emphasizing informative spatial and channel-wise features, with only a negligible increase in computational cost. Experiment 3 investigates the independent effect of the C2f_DCNv4 module. Compared with YOLOv8n, C2f_DCNv4 increases Precision from 0.9264 to 0.9376, while mAP@0.5 remains comparable (0.9507 vs. 0.9477). This indicates that although deformable convolution improves the model’s ability to capture complex geometric variations and enhance feature localization, its standalone integration does not consistently improve all evaluation metrics. Meanwhile, the parameter number and computational complexity increase from 3.01 M and 8.2 G to 5.63 M and 21.1 G, respectively, due to the additional deformable convolution operations. Therefore, the effectiveness of C2f_DCNv4 should be considered in terms of feature extraction capability and computational cost rather than only standalone accuracy improvement.

Experiment 4 evaluates the individual contribution of the Slide Loss. Compared to the baseline, it slightly improves mAP@0.5 from 0.9477 to 0.9486 and mAP@0.5:0.95 from 0.6120 to 0.6201 without introducing any additional parameters or computational overhead. Subsequently, Experiment 5 examines the independent influence of the P2 small object detection layer. The introduction of P2 significantly improves detection performance, increasing mAP@0.5 from 0.9477 to 0.9697 and Recall from 0.8927 to 0.9323. In particular, the considerable improvement in Recall indicates that the P2 layer effectively enhances the detection capability for small-scale targets. Although the computational complexity increases from 8.2 G to 14.5 G due to the additional high-resolution feature extraction pathway, the parameter number decreases to 2.01 M, suggesting a more efficient parameter utilization strategy.

**Figure 6:**
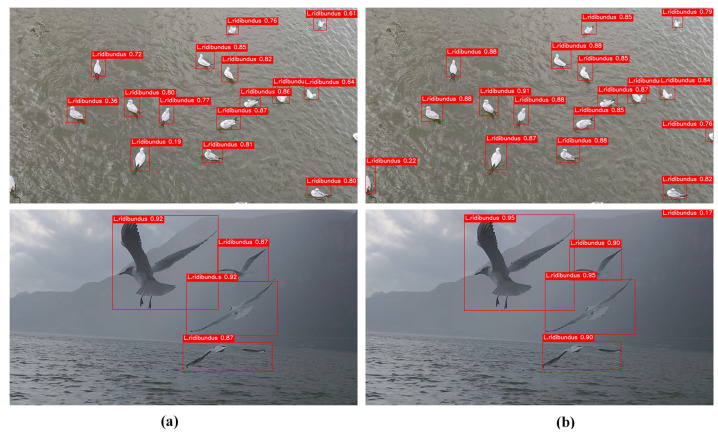
Comparison of detection capability between the YOLOv8n model and the CDSP2-YOLOv8n model. The left side (**a**) shows the performance of the original network, while the right side (**b**) displays the improved performance.

Furthermore, combination experiments were performed to investigate the synergistic effects among different modules. Experiment 6 combines CA and C2f_DCNv4, achieving improvements in Precision compared with the baseline model, while Experiment 7 further incorporates Slide Loss and obtains higher mAP@0.5:0.95 (0.6559) and Precision (0.9481), demonstrating that the optimization of feature representation and training objectives can complement each other. Finally, Experiment 8 integrates all proposed components and obtains the best overall performance, achieving mAP@0.5 of 0.9705, mAP@0.5:0.95 of 0.6557, Precision of 0.9685, and Recall of 0.9496. Figure 6 presents the detection comparison results between CDSP2-YOLOv8n and the baseline model, demonstrating the superior detection performance of the proposed method.

These results indicate that no single module contributes uniformly to all evaluation metrics. Instead, the final performance improvement of CDSP2-YOLOv8n results from the complementary effects of enhanced feature representation (CA and C2f_DCNv4), improved optimization strategy (Slide Loss), and strengthened small-object detection capability (P2). Although the integration of these modules introduces additional computational requirements, the substantial improvements in detection accuracy, particularly in reducing missed detections and false positives under complex backgrounds, justify the additional computational cost.

### 3.5. Comparison Experiment

To comprehensively evaluate the detection performance of the proposed CDSP2-YOLOv8n model, we compared it with several advanced object detection methods, including a range of representative general-purpose detectors such as Faster R-CNN [44], RT-DETRv2-R18 [45], YOLOv7-tiny [35], CenterNet [46], YOLO-Master [47], YOLOv13n [48], and YOLO26n [38]. Furthermore, considering the bird detection task investigated in this study, we additionally introduced several specialized small-object bird detection models from previous studies, including YOLOv7^Birds^ [25],YOLOv8-p2 [27], and an improved YOLOv9 model [26] incorporating AKConv, CAM, and AFF, for comparative evaluation. These comparisons were conducted to comprehensively verify the superiority of CDSP2-YOLOv8n in complex bird detection scenarios.

As shown in Table 4, CDSP2-YOLOv8n achieved the second-best overall detection performance among all evaluated models, obtaining an mAP@0.5 of 97.05%, an mAP@0.5:0.95 of 65.57%, a Precision of 96.85%, and a Recall of 94.96%. The YOLOv9-based model integrating AKConv, CAM, and AFF achieved slightly higher accuracy metrics, with an mAP@0.5 of 97.15% and an mAP@0.5:0.95 of 66.09%. However, this improvement was achieved at the expense of substantially increased model complexity, with 51.81 M parameters and 120.1 GFLOPs. In contrast, CDSP2-YOLOv8n requires only 3.09 M parameters and 27.5 GFLOPs, while maintaining comparable detection accuracy. These results demonstrate that CDSP2-YOLOv8n provides a more favorable balance between detection performance and computational efficiency. Among the general-purpose detectors, Faster R-CNN exhibited the lowest detection performance, achieving an mAP@0.5 of only 74.67% and a Precision of 54.32%. This indicates that conventional two-stage detection frameworks may have limitations in identifying small and densely distributed targets such as *L. ridibundus*. In comparison, single-stage detectors based on the YOLO architecture generally achieved better performance, demonstrating stronger adaptability for real-time small-object detection tasks. YOLOv13n achieved competitive performance with an mAP@0.5 of 96.41% and an mAP@0.5:0.95 of 65.01%, but its Precision and Recall remained lower than those of CDSP2-YOLOv8n.

From the perspective of model efficiency, YOLO26n achieved the lowest parameter count (2.51 M) and computational complexity (5.8 GFLOPs), demonstrating excellent lightweight characteristics. YOLO-Master and YOLOv13n also exhibited relatively low computational costs. However, these lightweight models showed relatively lower detection performance compared with CDSP2-YOLOv8n. Specifically, although YOLO26n requires only 5.8 GFLOPs, its mAP@0.5 and Recall were 96.00% and 91.40%, respectively, which were inferior to those of CDSP2-YOLOv8n. Compared with heavier models, CDSP2-YOLOv8n significantly reduces computational requirements while preserving high detection accuracy. For example, compared with Faster R-CNN and RT-DETRv2-R18, CDSP2-YOLOv8n reduces the parameter number by 133.91 M and 16.94 M, respectively, while decreasing computational complexity by 342.7 GFLOPs and 32.6 GFLOPs.

Furthermore, compared with specialized bird detection models, CDSP2-YOLOv8n also demonstrates competitive performance. YOLOv7^Birds^ achieved an mAP@0.5 of 96.87% and an mAP@0.5:0.95 of 65.33%, while YOLOv8-p2 obtained relatively lower performance. Although the YOLOv9-based model achieved marginally higher detection accuracy, its computational cost was approximately 4.4 times higher than that of CDSP2-YOLOv8n. Therefore, CDSP2-YOLOv8n achieves a better trade-off between accuracy and efficiency, which is particularly advantageous for practical bird monitoring applications.

Overall, the proposed CDSP2-YOLOv8n achieves the second-best detection performance among all compared models while maintaining relatively low computational complexity. It effectively enhances feature representation and detection capability for small objects in complex backgrounds, while avoiding a substantial increase in model complexity. These results demonstrate that CDSP2-YOLOv8n achieves a favorable balance between detection accuracy and computational efficiency, providing an efficient and reliable solution for small-object detection of *L. ridibundus*.

### 3.6. Results of Target Tracking and Counting

The performance of a MOT algorithm is strongly influenced by the detection accuracy of the object detector. To evaluate the tracking performance of different trackers, the proposed CDSP2-YOLOv8n was used as the object detector and combined with SORT [13], DeepSORT [15], OC-SORT [49], BoT-SORT [50], and ByteTrack [16], respectively. For tracking evaluation, 20 video sequences of *L. ridibundus* were manually annotated to generate trajectory ground truth. Each gull was assigned an individual identity ID, and its bounding box coordinates were recorded frame-by-frame throughout the entire sequence. Specifically, each gull was continuously tracked across consecutive frames, and the same identity ID was maintained throughout the entire trajectory. When an individual temporarily disappeared due to occlusion or moved out of the field of view, its original ID was retained if the individual could be reliably re-identified in subsequent frames. For newly appearing individuals, new identity IDs were assigned. The resulting annotations consisted of frame-level bounding boxes and corresponding identity labels, which were used as reference trajectories for evaluating tracking performance.

Table 5 shows that the combination of CDSP2-YOLOv8n and ByteTrack achieved the best overall tracking performance among all the evaluated methods, with a MOTA of 89.7%, a MOTP of 83.5%, an IDF1 of 87.5%, an MT of 82.1%, an ML of 13.8%, and 76 IDS. Although its inference speed of 21.4 FPS was slightly lower than the 22.5 FPS achieved by CDSP2-YOLOv8n combined with SORT, it still demonstrated superior overall tracking accuracy and robustness across the 20 test videos, reflecting the best comprehensive performance. These results indicate that the CDSP2-YOLOv8n + ByteTrack combination achieves improved tracking accuracy while maintaining high computational efficiency of MOT and counting for *L. ridibundus*.

**Figure 7:**
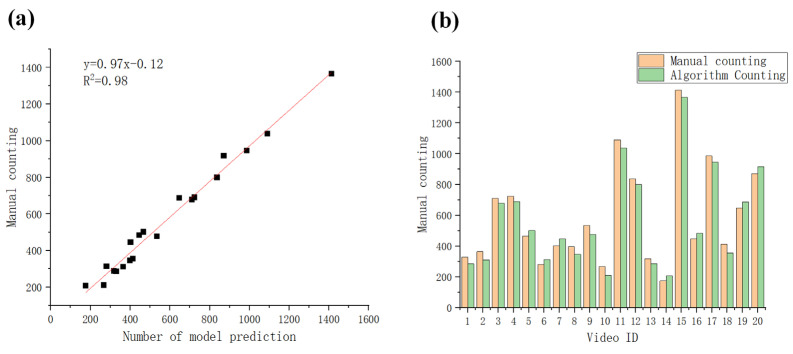
Comparison of manual and algorithmic counting results. (**a**) Manual counting and model prediction fit. (**b**) Algorithmic counting results.

Finally, the counting results obtained using the optimal CDSP2-YOLOv8n + ByteTrack framework were compared with the manual counting results provided by experienced observers. The observers manually reviewed all 20 test video sequences using the entire video frame as the counting region and counted each *L. ridibundus* individual only once according to its appearance and trajectory continuity within the field of view. For video segments involving occlusion, overlapping individuals, or uncertain counting events, the corresponding segments were replayed and examined frame by frame. Any inconsistent counting results were resolved by rechecking the relevant video segments, and the final manual counts were used as the reference standard. As shown in Figure 7, the proposed method achieved a coefficient of determination (*R*^2^) of 0.98285 and a counting accuracy of 91.58%. The counting accuracy was calculated according to Equation (14):

(14) $$\begin{array}{c} Counting\;Accuracy=\left(1-\frac{\sum_{i=1}^{N}\left|C_{i}^{alg}-C_{i}^{man}\right|}{\sum_{i=1}^{N}C_{i}^{man}}\right)\times 100\% \end{array}$$

where $C_{i}^{alg}$ denotes the counting result obtained by the algorithm for the ith video sequence, $C_{i}^{man}$ denotes the corresponding manual counting result obtained by experienced observers, and *N* denotes the total number of video sequences used for counting evaluation.

These findings demonstrate that the proposed framework (CDSP2-YOLOv8n + ByteTrack) achieves a high level of agreement with manual counting results and has promising potential for practical applications.

### 3.7. Application Design

**Figure 8:**
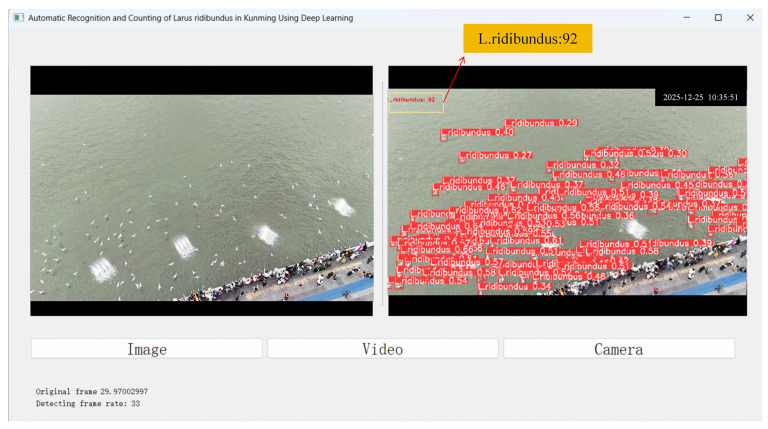
Interface and monitoring results of the detection system.

To implement the proposed method for MOT and counting of *L. ridibundus*, the trained CDSP2-YOLOv8n detector was integrated with the ByteTrack multi-object tracker and deployed on a local computer to establish an intelligent monitoring and counting system for *L. ridibundus*, as illustrated in Figure 8. The system can be integrated with video surveillance devices to enable real-time detection, tracking, and counting of *L. ridibundus*.

The experimental results demonstrate that the system can continuously track *L. ridibundus* across consecutive frames and accurately count individuals in video sequences. These findings further validate the effectiveness of the proposed method for the detection, tracking, and counting of *L. ridibundus*, providing reliable technical support for population monitoring, abundance estimation, and conservation management of this species.

## 4. Discussion

This study proposes an advanced DL framework for the automated identification and tracking of *L. ridibundus*. By integrating deeper convolutional neural networks with efficient feature-extraction mechanisms, the framework achieves high-precision multi-object tracking and counting of *L. ridibundus*. The CDSP2-YOLOv8n model is combined with the ByteTrack algorithm to enable continuous tracking of *L. ridibundus* in video streams, effectively addressing challenges such as small target size and rapid movement while maintaining high tracking accuracy and stability. Furthermore, a DL-based system was developed for the automated identification and counting of *L. ridibundus*. This technology not only improves the monitoring of *L. ridibundus* populations but also provides essential technical support for conservation efforts, substantially enhancing monitoring efficiency and accuracy.

The proposed technical framework effectively addresses the challenges associated with small object detection and occlusion in identifying and counting *L. ridibundus*; however, several limitations persist. First, under extreme conditions, variations in lighting can negatively impact the model’s accuracy and robustness in recognizing *L. ridibundus*. Furthermore, image quality may impede the model’s recognition performance. Finally, the inference speed of 21.4 FPS was measured on a workstation equipped with an RTX 3080 GPU, and its performance on edge devices may therefore be limited. Notably, despite these limitations, the proposed technical framework exhibits significant potential for identifying and counting *L. ridibundus*, owing to its high adaptability and scalability.

Future research will primarily focus on addressing these limitations, which include (i) developing a large-scale multi-object recognition and tracking dataset for *L. ridibundus* that encompasses diverse and complex environments to enhance the model’s generalization ability and robustness under challenging conditions; (ii) implementing image enhancement techniques or super-resolution reconstruction algorithms to improve detail features in images captured from long distances or at low resolutions, thereby minimizing background interference and increasing the model’s accuracy in recognizing and distinguishing blurred targets and species with similar body sizes; (iii) investigating more advanced DL architectures and multi-modal monitoring methods, such as integrating vision-language models, graph neural networks, or UAV-assisted collaborative monitoring technologies, to further optimize the stability of multi-target tracking algorithms in complex occlusion environments, while also attempting to extend this framework to the dynamic population monitoring of other bird species or wildlife; and (iv) optimizing the model for deployment and practical application on edge devices. Addressing these limitations will enable the further application of the CDSP2-YOLOv8n + ByteTrack framework proposed in this study for the population monitoring of *L. ridibundus*.

## 5. Conclusions

This study introduces a novel, efficient, and precise automated method for recognizing and counting *L. ridibundus* in Kunming. This method effectively addresses the challenges associated with detecting small objects and managing occlusion during the identification and enumeration of *L. ridibundus*, thereby offering a scalable and effective tool for population monitoring and conservation research. By integrating CDSP2-YOLOv8n with ByteTrack technology, this approach enhances both monitoring efficiency and accuracy, minimizes human interference, and provides a scalable technical solution for long-term field monitoring in complex environments. As technological advancements continue, this method’s significance in *L. ridibundus* population monitoring, ecological conservation, and related fields is expected to grow, thereby providing substantial support for behavioral analysis, health assessments, and ecological studies of this species.

## Figures and Tables

**Table 1:** Comparison of training results with attention mechanism.

Neural Network Model	mAP@0.5	mAP@0.5:0.95	Precision	Recall	Params (M)	GFLOPs (G)
YOLOv8n	0.9477	0.6120	0.9264	0.8927	3.01	8.2
YOLOv8n_CA	0.9557	0.6372	0.9357	0.9093	3.05	8.3
YOLOv8n_Swin Transformer	0.9551	0.6327	0.9343	0.9046	3.37	18.6
YOLOv8n_GAM	0.9571	0.6501	0.9407	0.9077	4.65	9.5

**Table 2:** Ablation study of small object detection layer.

Neural Network Model	mAP@0.5	mAP@0.5:0.95	Precision	Recall	Params (M)	GFLOPs (G)
YOLOv8n	0.9477	0.6120	0.9264	0.8927	3.01	8.2
YOLOv8n_P2345	0.9665	0.6369	0.9313	0.9283	2.93	12.4
YOLOv8n_P234	0.9697	0.6442	0.9366	0.9323	2.01	11.6

**Table 3:** Ablation study results.

Number	CA	C2f_Dcnv4	Slide	P2	mAP@0.5	mAP@0.5:0.95	Precision	Recall	Params (M)	GFLOPs (G)
1	×	×	×	×	0.9477	0.6120	0.9264	0.8927	3.01	8.2
2	√	×	×	×	0.9557	0.6372	0.9357	0.9093	3.05	8.3
3	×	√	×	×	0.9507	0.6313	0.9376	0.8984	5.63	21.1
4	×	×	√	×	0.9486	0.6201	0.92809	0.8998	3.01	8.2
5	×	×	×	√	0.9697	0.6442	0.9366	0.9323	2.01	14.5
6	√	√	×	×	0.9561	0.6265	0.9404	0.9006	5.67	21.2
7	√	√	√	×	0.9619	0.6559	0.9481	0.9145	5.67	21.2
8	√	√	√	√	0.9705	0.6557	0.9685	0.9496	3.09	27.5

Note: The symbol “√” denotes the use of the corresponding component, while “×” denotes the absence of the corresponding component.

**Table 4:** Performance comparison of CDSP2-YOLOv8n and other classical models.

Neural Network Model	mAP@0.5	mAP@0.5:0.95	Precision	Recall	Params (M)	GFLOPs (G)
Faster R-CNN	0.7467	0.5340	0.5432	0.7996	137	370.2
RT-DETRv2-R18	0.9333	0.6382	0.9137	0.9115	20.03	60.1
YOLOv7-tiny	0.9608	0.5631	0.9453	0.9322	6.01	13.0
Center Net	0.9208	0.6100	0.9752	0.7064	32.67	70.2
YOLO-Master	0.9440	0.6140	0.9220	0.8930	2.66	8.6
YOLOv13n	0.9641	0.6501	0.9310	0.9170	2.64	6.4
YOLO26n	0.9600	0.6480	0.9280	0.9140	2.51	5.8
YOLOv7^Birds^	0.9687	0.6533	0.9649	0.9409	41.53	60.15
YOLOv8-p2	0.9640	0.6301	0.9344	0.9180	2.93	12.4
YOLOv9 + AKConv + CAM + AFF	0.9715	0.6609	0.9693	0.9505	51.81	120.1
CDSP2-YOLOv8n	0.9705	0.6557	0.9685	0.9496	3.09	27.5

**Table 5:** Comparison of target tracking network accuracy.

Algorithm	MOTA (%)	MOTP (%)	IDF1 (%)	MT (%)	ML (%)	IDS	FPS (Frames/s)
+SORT	86.6	76.3	80.2	69.5	23.2	169	22.5
+DeepSORT	87.2	78.4	82.7	72.1	20.8	124	18.2
+OC-SORT	87.3	80.5	83.2	74.5	18.7	111	19.5
+BoT-SORT	88.3	82.2	85.1	78.4	15.9	97	20.7
+ByteTrack	89.7	83.5	87.5	82.1	13.8	76	21.4

## Data Availability

The raw and processed data presented in this study are available on reasonable request from the corresponding author, Jiajin Zhang, or the first author, Yonglin Che.

## Abbreviations

The following abbreviations are used in this manuscript:

*L. ridibundus*	*Larus ridibundus*
DL	Deep learning
MOTA	Multiple Object Tracking Accuracy
MOTP	Multiple Object Tracking Precision
MT	Mostly Tracked
ML	Mostly Lost
IDS	Identity Switches
CV	Computer vision
AI	Artificial intelligence
CA	Coordinate attention
FPN	Feature Pyramid Network
PAN	Path Aggregation Network
SPP	Spatial pyramid pooling
IoU	Intersection over Union
MOT	Multi-object tracking
ID	Identity
NMS	Non-maximum suppression
